# Influence of Injection Application on the Sol–Gel Phase Transition Conditions of Polysaccharide-Based Hydrogels

**DOI:** 10.3390/ijms222413208

**Published:** 2021-12-08

**Authors:** Anna Rył, Piotr Owczarz

**Affiliations:** Department of Chemical Engineering, Lodz University of Technology, 90-924 Lodz, Poland; piotr.owczarz@p.lodz.pl

**Keywords:** injectable hydrogels, thermoinduced gelation, HPC, chitosan, orthokinetic aggregation, perikinetic aggregation, shear-induced molecules deformation

## Abstract

Polysaccharide matrices formed via thermoinduced sol–gel phase transition are promising systems used as drug carriers and minimally invasiveness scaffolds in tissue engineering. The strong shear field generated during injection may lead to changes in the conformation of polymer molecules and, consequently, affect the gelation conditions that have not been studied so far. Chitosan (CS) and hydroxypropyl cellulose (HPC) sols were injected through injection needles (14 G–25 G) or sheared directly in the rheometer measuring system. Then the sol–gel phase transition conditions were determined at 37 °C using rheometric, turbidimetric, and rheo-optical techniques. It was found that the use of low, respecting injection, shear rates accelerate the gelation, its increase extends the gelation time; applying the highest shear rates may significantly slow down (HPC) or accelerate gelation (CS) depending on thixotropic properties. From a practical point of view, the conducted research indicates that the use of thin needles without preliminary tests may lead to an extension of the gelation time and consequently the spilling of the polymeric carrier before gelation. Finally, an interpretation of the influence of an intensive shear field on the conformation of the molecules on a molecular scale was proposed.

## 1. Introduction

Recently, in the biomedical engineering field, a growing interest in innovative methods of treatment and regeneration has been observed [1]. Injectable hydrogels [2,3,4] obtained from natural and synthetic polymers may provide a very promising solution. Interest in these systems results from their lower invasiveness compared to implantation scaffolds, reduced risk of infection and scarring, as well as better filling of difficult, irregular defects [5]. Moreover, due to their similarity to the extracellular matrix [6,7], polymer hydrogels are an excellent scaffold for cell adhesion and transport of active substances such as growth factors.

Depending on the form of the formulation before and after administration, as well as the mechanism of creating an unlimited polymer structure, three groups of materials are distinguished: shear thinning injectable gels, self-assembling suspensions of solid particles, and in situ gelling liquids [8]. The latter creates a three-dimensional polymer matrix directly in the body as a result of the sol–gel phase transition induced by one or a combination of different stimuli [9,10], the most common temperature changes [11,12,13]. Before and during the application, such systems remain in a liquid sol form, which, according to most researchers, should ensure the possibility of their injection [4]. However, there is no instrumental research confirming this thesis. Additionally, thermosensitive injectable hydrogels should be characterized by a lower critical solution temperature (LCST) [14,15] which would ensure the formation of a lattice upon heating. The most frequently discussed materials in the literature that fulfill this requirement are systems made of polysaccharides like chitosan [16,17,18], cellulose derivatives such as HPC [19,20,21], HPMC [22,23], and synthetic polymers, e.g., PNIPAAM [24] and PLGA [25]. Such systems are often subject to further modifications leading to the improvement of mechanical properties [18,26,27].

The injection application, although minimally invasive for the patient, involves the flow of the polymer sol through the capillary under high shear rates conditions [28,29]. Rył and Owczarz [28] showed that during the flow of Newtonian liquid injected at a typical manual injection rate of 1 mm/s [30,31], the observed shear rate ranges from approx. 200 to over 43,000 s^−1^ (depending on the needle diameter) or even 55,000 s^−1^ taking into account the strongly shear-thinned nature of polymer systems. As a consequence, high shear stresses are generated which may affect the spatial conformation of the polymer molecules including their arrangement along the streamlines The previous authors’ research [32] has shown that the spatial conformation of biopolymer molecules will strongly depend on the applied shear field, its direction and magnitude.

Despite the phenomenon of ordering polymer chains under the influence of the shear field, which has been repeatedly reported [33,34,35], the results of studies discussing the impact of these changes on the possibility of creating an unlimited lattice, gelation conditions, and the mechanism have not been published so far. This discussion is most often limited to the shear-induced phase separation of polymer systems, the solidification of which is observed under the influence of the shear field and the production of the necessary mechanical work [36]. In this case, no other trigger is observed that could lead to phase separation. In addition, the cited studies on polymer systems mostly use moderate shear rate values (up to 100 s^−1^), which do not correspond to the values during the injection. Dunderdale and co-authors [37] showed that in the case of metastable aqueous PEO solutions, the hydration sheath ruptures under the influence of the shear field. Consequently, this leads to the formation of hydrophobic interactions and proper crystallization.

The shearing of colloidal systems such as emulsions and silicas can also lead to aggregation in the orthokinetic regime, which accelerates the formation of the spatial structure compared to aggregation limited only by stochastic Brownian motion by lowering the energy barrier. Moreover, under the influence of the velocity gradient, the structures may be reorganized, including their fragmentation and changing the cluster dimension by breaking it up [38,39,40,41,42].

Shear effect under injection conditions have been discussed in detail for proteins [43]. It has been proven in numerous studies that flow-induced structural changes [44], including aggregation caused by destabilization of the native conformation of proteins, exposing functional groups leading to the generation of hydrophobic interactions could be observed [45,46]. Consequently, these changes can lead to the reversible and irreversible deactivation of enzymes. The former is triggered by the deformation of the molecules along the shear field, whereas the latter is caused by the destruction of the tertiary protein structure [47]. Overall, in the case of protein systems, this effect depends on the intensity of the shear field as well as its duration.

This study aims to investigate the effect of the short-term flow of polysaccharide sol under high shear rates conditions on the possibility of creating an unlimited polymer network. It has been hypothesized that shear-induced changes in molecular conformation, including fragmentation, occurring during an injection would negatively affect the aggregation kinetics or even completely prevent the lattice formation. Simultaneously, the mechanical energy generated as a result of shearing may constitute an additional source of energy supplied to the system to induce an endothermic sol–gel phase transition.

## 2. Results

### 2.1. Preliminary Studies of Hydroxypropyl Cellulose Colloidal Suspension

**Figure 1:**
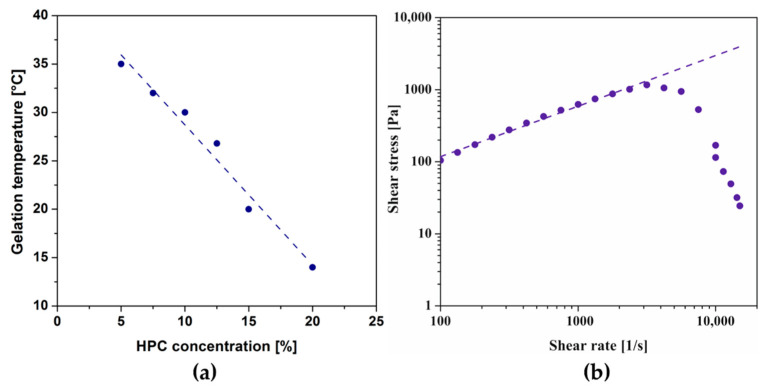
(**a**) Dependence of gelation temperature of HPC on polymer concentration. (**b**) Flow curve of a 10% HPC solution at 20 °C.

Due to the key requirement of thermosensitive injectable scaffolds forming a three-dimensional lattice in the human body, the polymer concentration was first optimized, which strongly determines the LCST value. Based on the obtained results under non-isothermal conditions, the gelation point was defined at the temperature at which the storage modulus G′ and loss modulus G′′ reached the same value. Figure 1a shows the dependence of the gelation temperature on the polymer concentration. A 10% HPC solution was selected for further research, which reached the phase transition point at a temperature of approx. 30 °C, thus ensuring the appropriate kinetics of forming the spatial structure and preventing gelation during the application.

Based on the obtained flow curves presented in Figure 1b, it has been shown that the 10% colloidal HPC suspension exhibits non-Newtonian behavior. The determined degree of shear thinning is lower (*n* = 0.7) than in the case of chitosan systems (*n* = 0.44) [28]. It is worth noting that after exceeding the value of the shear rate equal to 3160 s^−1^, the values of the shear stress decrease, which is inconsistent with the measurement theory. It may suggest that permanent structural changes occur in the tested system, and it is impossible to unequivocally assess the properties above the indicated shear rate.

### 2.2. Instrumental Injectability Tests

**Figure 2:**
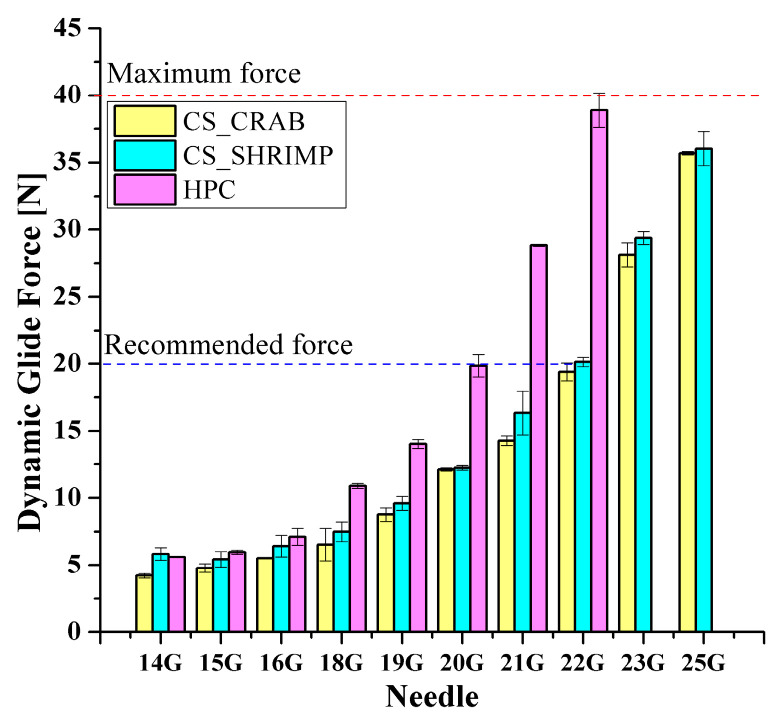
Effect of injection needle size on the value of DGF force for crab-derived chitosan (CS_CRAB) [28], shrimp-derived chitosan (CS_SHRIMP), and HPC systems.

Figure 2 shows the results of instrumental injectability tests. It is evident that, regardless of the origin of the chitosan, both tested systems are injectable with 14 G–25 G needles, and the higher molecular weight of the polymer causes a slight increase in the value of the dynamic glide force DGF. The value recommended for injection is significantly exceeded in the case of the use of 23 G and 25 G needles, while not reaching the value of 40 N, considered the maximum possible force to be generated based on panel tests [48]. In the case of the HPC-based system, using a typical crosshead speed corresponding to manual injection equal to 1 mm/s, the value of the recommended dynamic force DGF does not exceed 20 N when using needles larger than 20 G. When smaller needles are used, this value increases rapidly and reaches the maximum allowable force for the 22 G needle. Attempts at instrumental injectability analysis for smaller needles were unsuccessful due to the allowable load on the apparatus. Simultaneously, a force value of 40 N is considered the maximum force value that can be generated during an injection application by medical staff [49]. This means that compared to chitosan systems, the HPC sol is more difficult to inject due to its less shear thinning nature and higher apparent viscosities. Nevertheless, with the use of sufficiently large needles (preferably not smaller than 19 G), the injection should be successful.

### 2.3. Kinetics of the Sol–Gel Phase Transition after Shear

**Figure 3:**
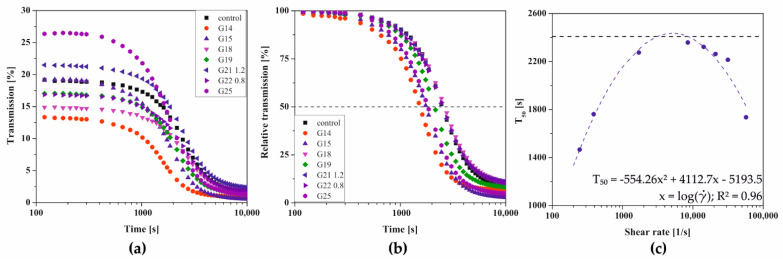
Kinetics of changes in the parameters of (**a**) transmission and (**b**) relative transmission versus time. (**c**) The dependence of the T_50_ time on the shear rate during injection.

The influence of the injection application on the conditions of aggregation limited solely by stochastic Brownian motion, the so-called perikinetic aggregation regime, was determined firstly based on changes in a transmitted signal versus time—Figure 3a. It can be seen that regardless of the needle used during the injection preceding the actual sol–gel phase transition, the experimental curves assume the shape typical of progressive aggregation accompanied by an increase in opacity limiting light transmission. Similar changes were observed in the case of cellulose derivatives such as HPC [20,21], MC, and HPMC [23] as well as in the authors’ earlier works on chitosan systems [32,50]. In order to quantify the phase transition conditions, the experimental data were normalized to obtain the relative transmission parameter (Figure 3b); the time in which this parameter reached 50% determines the characteristic time of the phase transition [20,21,50].

The dependence of the T_50_ time on the shear rate occurring in injection needles is shown in Figure 3c. Based on the obtained data, it can be seen that regardless of the needles used, the initial, short-term shearing interval accelerates the aggregation, compared to the control sample when the characteristic time was 2409 s. In the case of low shear rates, concerning injection, the gelation time was slightly more than 50% of the control time. With the use of smaller needles, which increase the shear rate, the critical time increases until the maximum value for the 21 G needle (approx. 7380 s^−1^) is reached, and then decreases again. The critical value of the shear rate of $\dot{\gamma }$ = 5130 s^−1^, the exceeding of which causes the observed changes, was determined as the extreme of the function approximating the experimental data, which, due to the course of the experimental points, took the form of a second-degree polynomial.

**Figure 4:**
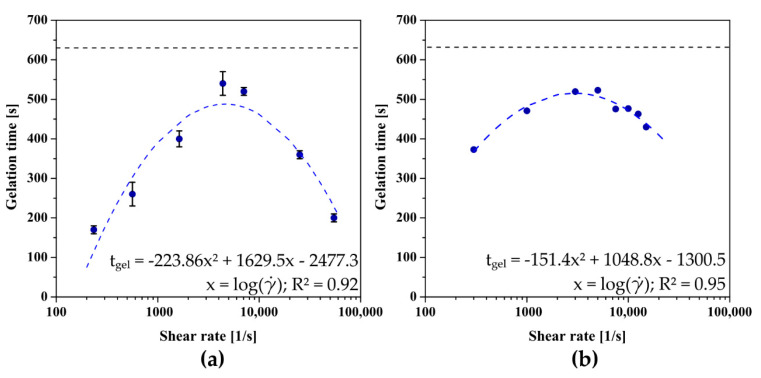
The dependence of the gelation time on the shear rate occurring during the flow through (**a**) the needle or (**b**) pre-shear realized directly in the measuring system of the rheometer for crab-derived chitosan.

Due to the significant influence of the aggregation regime on the gelation conditions, which was demonstrated in the previous work of the authors [32], the conditions for forming a three-dimensional polymer network in the rheometer measurement system were determined. As in the case of aggregation in the perikinetic regime, injecting chitosan sol through any needle significantly accelerates the gelation point (Figure 4a), determined at the point where the values of dynamic modules are equalized. Injection through the largest of the needles used (14 G) leads to a nearly four-fold reduction in the time after which the intersection of the storage modulus G′ and the loss modulus G′′ curves is observed. It is again visible that with the increase of the shear rate the gelation time is lengthened until the critical shear rate value of 4361 s^−1^ is reached; then the time needed to change the dominant properties of the chitosan sol (from viscous to elastic) is shortened again.

It should be noted that due to the use of classic literature methods for determining the gelation time, it is not possible to directly compare the obtained results for different aggregation regimes. This is due to the fact that both methods used define points located in other areas of the generalized dynamics curve of characteristic quantity changes during the sol–gel phase transition [50].

Despite the best mapping of the actual conditions of forming the polymer matrix after injection, the application of the above methodology is associated with certain technical inconveniences and the necessity to use, among others, an infusion pump with precisely set flow parameters corresponding to manual injection. For this reason, it was decided to map the shear conditions directly in the rheometer measuring system by using an initial rotational interval before the proper measurement of the thermoinduced sol–gel phase transition kinetics. As shown in Figure 4b, the dependence of the gelation time as a function of the applied shear rate is again a non-monotonic function with clear extremes occurring at the shear rate equal to $\dot{\gamma }$ = 2908 s^−1^. Despite the satisfactory mapping of the experimental data and consequently shape of the approximating function, when using the initial shear interval, slight differences are observed due to the way the sample is sheared. Moreover, the critical shear rate value determined is significantly lower than the other two obtained after a conventional injection. This may be due to the inability to apply shear rates above 15,000 s^−1^ because of rheometer limitations, which consequently prevents obtaining more measurement data in the range of high shear rates.

### 2.4. Comparison of Aggregation Conditions in the Regimes of Perikinetic and Orthokinetic after Short-Term Shear

**Figure 5:**
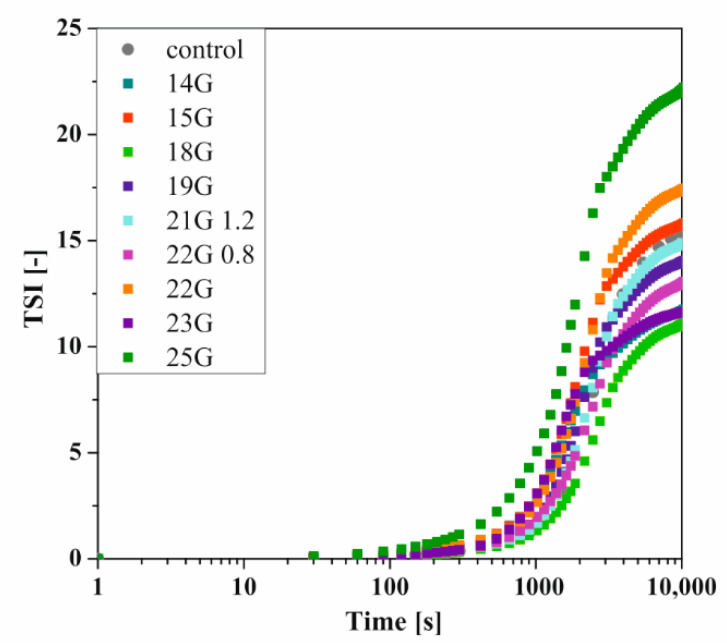
Kinetics of changes of the TSI parameter versus time depending on the needle used for crab-derived chitosan.

As mentioned above, the use of classical methods for determining the gelation time or the characteristic time of turbidimetric measurements prevents quantitative comparison of aggregation conditions. It is known that the method commonly used in rheometric measurements, based on the equalization of the values of the dynamic modules G′ and G′′, only defines the point at which the change of the dominant properties of the medium from viscous to elastic is observed. This point indicates the beginning of the fast gelation area, exceeding which is associated with a rapid increase in the value of both dynamic modules-even by several decades [51]. Whereas the T_50_ time, i.e., the time when the relative transmission reaches 50%, will be observed in the half of the fast gelation region. This means that completely different states of the structure would be compared, and the time T_50_ itself, by definition, will be longer. Owczarz et al. [50] suggested the possibility of replacing the relative transmission value by the total destabilization index TSI, the shape of which takes the form of the letter S, characteristic of dynamic changes of selected rheological or optical values during the gelation process. The influence of the injection needle used on the kinetics of changes of the TSI parameter versus time is presented in Figure 5.

**Figure 6:**
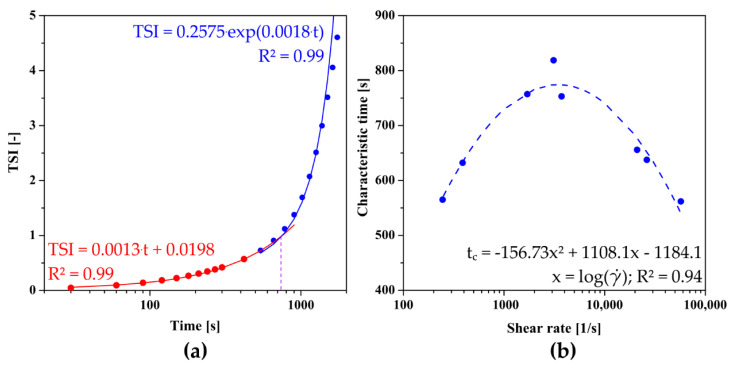
(**a**) An example of determining the characteristic time based on the proprietary method. (**b**) The dependence of the characteristic time determined from turbidimetric measurements on the shear rate observed in the needle for crab-derived chitosan.

In previous studies, for the quantitative analysis of the sol–gel phase transition condition using TSI curve, the authors proposed a method of determining the characteristic time in analogy to the one presented by Rwei [20] and Gosecki [21] in which the relative TSI reaches 50% [50], or a graphical method based on the intersection point of two lines passing through the induction area and the fast gelation region [32]. In order to take into account the kinetics of changes in the fast gelation region as well as to avoid one-point analysis, it was decided to further develop the above methods. For this purpose, the fast gelation region was described by an exponential function which in most theoretical models describes the area of rapid changes during progressive aggregation [50,52]. Contrary to the literature, the initial changes were described by a linear function, not by one averaged value of the parameter. As shown in Figure 6a, the point of intersection of both approximating functions determines the characteristic point of the phase transition. Figure 6b shows the dependence of the characteristic time on the shear rate, which is a non-monotonic function with a clear extreme occurring at the shear rate equal to $\dot{\gamma }$ = 3428 s^−1^.

**Figure 7:**
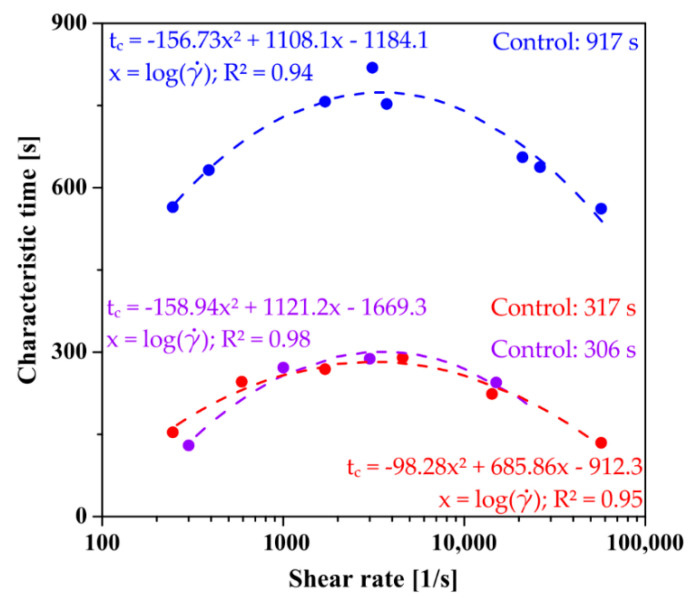
Comparison of the determined characteristic times of the phase transition as a function of shear rate for perikinetic aggregation (blue), orthokinetics preceded by injection (red), and ortokinetics preceded by an initial shear interval realized directly in the rheometer measuring system (violet).

The rheological data was described analogously; the analyzed value was the storage module G′ representing the formation of the polymer network. Regardless of the aggregation regime and the type of the applied shear field in the initial interval, the course of the experimental data (Figure 7) is identical to those determined based on classic literature methods. In all cases, the obtained characteristic times after pre-shear are shorter than those determined for the control measurements. In the case of aggregation in the orthokinetic regime, the critical value of the shear rate in the case of injection through the needle is $\dot{\gamma }$ = 3085 s^−1^, while when using the initial shear interval directly in the rheometer measurement system, the value is $\dot{\gamma }$ = 3366 s^−1^.

Based on the obtained data, it can be unequivocally stated that the gelation carried out in the rheometer measuring system proceeds much faster than that induced solely by stochastic Brownian motion. The determined times in the orthokinetic regime are almost three times shorter than those obtained for aggregation in the perikinetic regime. Thus, the obtained data are in line with literature reports for colloidal systems [38,39,40,41,42].

### 2.5. Aggregation Conditions of Shrimp Chitosan in the Peri- and Orthokinetic Regimes after Short-Term Shear

**Figure 8:**
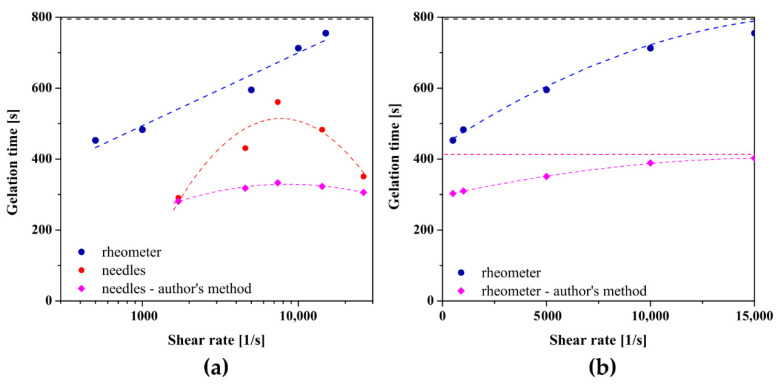
The dependence of gelation times on the shear rate presented on (**a**) logarithmic and (**b**) linear scale for shrimp-derived chitosan.

Figure 8 shows the results obtained for chitosan sol characterized by a higher molecular weight. In the case of studies conducted in the orthokinetic aggregation regime preceded by the injection of the experimental medium, a non-monotonic dependence of the gelation time on the shear rate was again observed, and the approximating function of the experimental data reaches its extreme at a shear rate of 7730 s^−1^. The longest gelation time equal to 561 s was obtained using a 21 G needle ($\dot{\gamma \;}$= 7380 s^−1^) in which the shear rate values during the flow are closest to the critical value, while for the control measurement it was the value of 798 s. Thus, the beneficial effect of injections on the sol–gel phase transition conditions was again observed. In the case of applying the initial shear interval directly in the measuring system of the rheometer, slightly different results were obtained. Although the application of an initial shear interval of any intensity accelerates the phase transition, the dependence of the determined gelation time on a log-linear scale is a straight line. It turns out that presenting the obtained results in a classical linear scale enables the interpolation of the experimental data with a second-degree polynomial, and the experimental data are arranged along one increasing curvature of the parabola. Based on the determined coefficients of the polynomial, the critical shear rate at which the extremum is predicted is $\dot{\gamma }$= 19,200 s^−1^. It should be noted that this value exceeds the measuring range of the device declared by the manufacturer; therefore, the obtained value must be approached with caution. Such a significant difference, compared to the results obtained for crab-derived chitosan, may result from the lower invasiveness of the initial shear interval directly in the rheometer measuring system, including the deviation from the Poiseuille flow.

The dependence of the characteristic time, determined based on the proprietary method, on the shear rate in the initial interval, and in particular, the effect of accelerating the gelation process as well as the course of experimental data, are consistent with the results obtained using the method of equalizing the values of dynamic modules. Based on the interpolation of the obtained results, the critical values of the shear rate were determined during injection through the needle and using the initial shear interval directly in the rheometer measuring system were 8589 s^−1^ and 16,375 s^−1^, respectively. As in the case of the analysis based on the literature methods of determining the phase transition point, the value of the critical shear rate for aggregation in the orthokinetic regime preceded by the rotational interval in the rheometer must be analyzed with great care due to the exceeding of the measuring range of the instrument.

**Figure 9:**
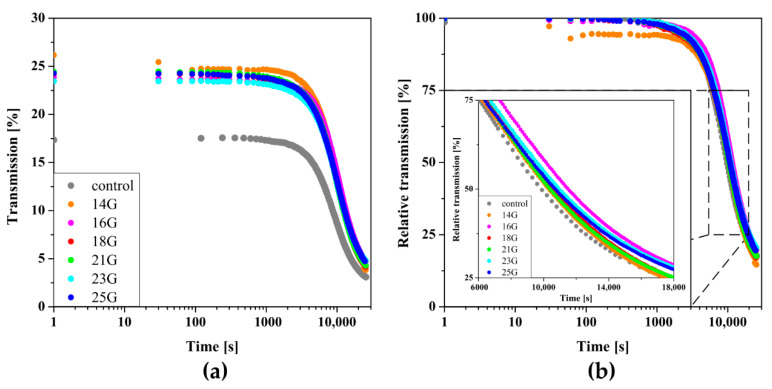
Kinetics of changes in the parameters of (**a**) transmission and (**b**) relative transmission versus time.

Figure 9 shows the results of measurements carried out in the perikinetic aggregation regime. Compared to the sol obtained from a lower molecular weight polymer, the characteristic decrease in transmission values associated with the phase transition was observed after a longer period, indicating a slower aggregation process leading to the formation of a three-dimensional polymer network. Based on the analysis of the change in the relative transmission parameter, it can be seen that the injection has a much smaller effect on the time when the T_50_ parameter reaches the value of 50%. Due to the slight differences of about 10% with such long characteristic times, it seems unjustified to analyze the dependence of the characteristic time on the shear rate occurring in the injection needle. Small changes in the experimental curves may result from the limiting effect of slow aggregation kinetics, which masks the influence of pre-shear.

**Figure 10:**
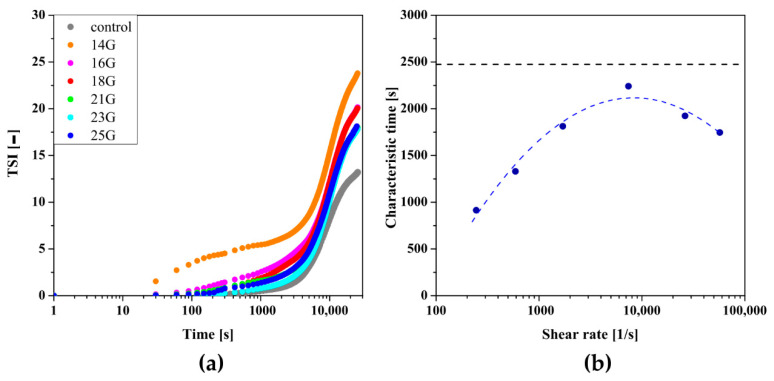
(**a**) Kinetics of the TSI parameter changes over time for shrimp-derived chitosan. (**b**) The dependence of the determined characteristic time on the shear rate observed during the injection.

As in the case of crab-derived chitosan, the change in the total destabilization index TSI (Figure 10a) of the system was analyzed to take into account changes in the both signals values of transmitted as well as those backscattered from colloidal particles. The obtained data were used for further analysis leading to the determination of the dependence of the characteristic time on the shear rate (Figure 10b), which again takes the form of a non-monotonic function with a clear extreme. In the case of the control measurement, the obtained characteristic time value was 2474 s; about 2.5 times longer than for crab chitosan and six times longer than for aggregation of shrimp chitosan in the orthokinetic regime. When the largest 14 G injection needle was used in which the flow takes place under conditions of low shear rates ($\dot{\gamma }\;$= 245 s^−1^), the value of the characteristic time was 914 s. The increase in shear rate due to the use of a smaller injection needle resulted in longer gelation, characteristic time until the extreme ($\dot{\gamma }=8320\;s^{-1}$) was reached with the 21 G needle. During the flow through this needle, the shear rate was 7380 s^−1^, and the characteristic time was 2241 s. The use of needles with a smaller diameter again shortens the characteristic time.

### 2.6. Aggregation Conditions of Hydroxypropyl Cellulose in the Peri- and Orthokinetic Regimes after Short-Term Shear

**Figure 11:**
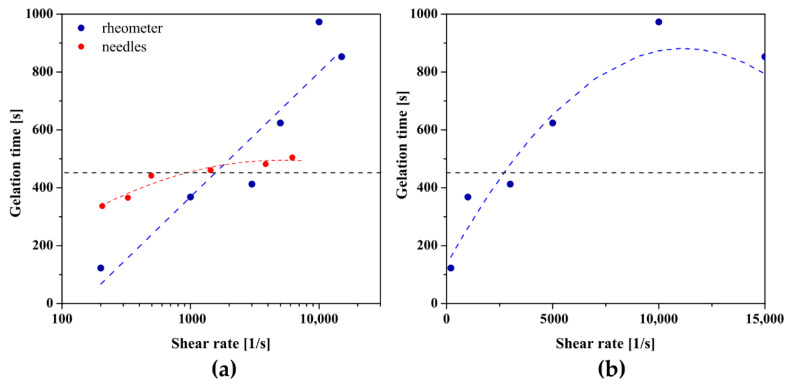
The dependence of gelation times on the shear rate presented on (**a**) logarithmic and (**b**) linear scale for HPC.

Figure 11 shows the results for the hydroxypropyl cellulose sol. It is visible that the use of large needles (14 G–16 G) accelerates the gelation, but the intensity of this phenomenon is weaker than in the case of chitosan systems. The use of an 18 G needle results in a gelation time almost identical to that in the measurements, 461 s and 458 s, respectively. Injection of the HPC sol through the smaller needles, i.e., 20 G and 21 G, increased the time needed to reach the damping factor value of tan(δ) = 1. Due to the inability to inject the medium through needles smaller than 21 G (Figure 2), it was impossible to determine the effect of applying a shear rate higher than 6200 s^−1^ on the gelation conditions. When using the initial rotational interval preceding the aggregation of the polymer system using a log-linear scale, a linear dependence of the gelation time on the value of the shear rate is visible. Applying shear below $\dot{\gamma }\;$= 1560 s^−1^ shortens the time needed to equalize the values of the storage G′ and the loss G′′ moduli, while the use of higher shear rates slows down the process significantly; in the extreme case, more than twice-for $\dot{\gamma }\;$= 10,000 s^−1^ gelation time was 973 s, while for the control-452 s. As in the case of shrimp chitosan, the presentation of the obtained data on a linear scale enables their interpolation with a second-degree polynomial and, consequently, the determination of the critical shear rate equal to $\dot{\gamma }\;$= 11,166 s^−1^. Due to the limitations of the rotational rheometer as well as the inability to inject HPC through small needles, it is not possible to unequivocally determine the behavior of the tested system using higher than critical shear rates.

**Figure 12:**
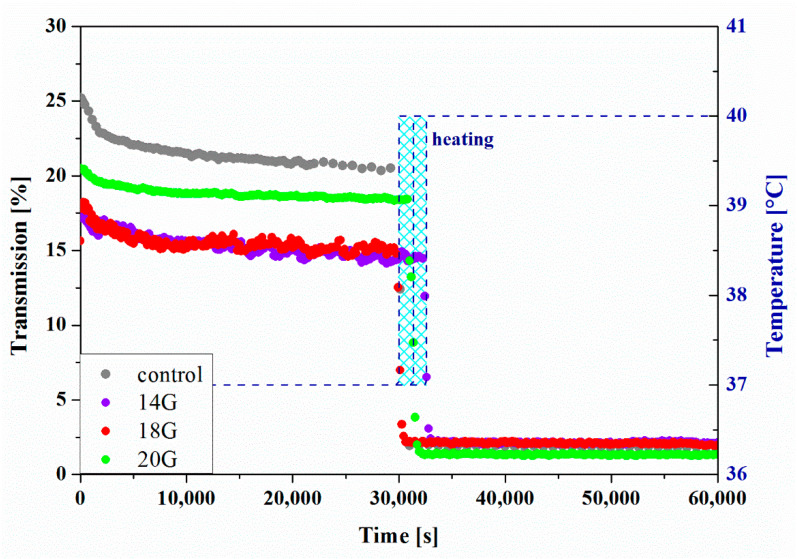
Kinetics of the transmission parameter changes versus time for HPC.

The results of the measurements carried out in the perikinetic aggregation regime are shown in Figure 12. Based on slight changes in the transmission values obtained at 37 °C, it can be stated that the gelation process does not occur. Only a change in the environmental conditions by heating the measuring chamber to 40 °C after about 8 h of measurement causes a rapid decrease in the value of the analyzed signal. Due to the inability to precisely control the heating rate, it was considered unjustified to conduct further quantitative analysis of the phenomenon. Simultaneously, based on the obtained results, it was unequivocally demonstrated that, despite the possibility of determining gelation times based on the equalization of the values of dynamic modules, aggregation limited only by stochastic Brownian motion does not occur during the measurement carried out under the same conditions, i.e., temperature.

### 2.7. Reo-Optical Analysis of Thixotropic Properties

Due to the significant influence of the shear field on the phase transition conditions of the investigated formulations, an attempt was made to determine the changes in thixotropic properties after injection using classical rheological techniques combined with a parallel small-angle light scattering analysis, the so-called Rheo-SALS technique [41,51,53]. When monitoring the dynamics of the sol–gel phase transition phenomenon induced by environmental stimuli, the key value in the analysis is the storage modulus G′ representing the elastic properties of the medium; its rapid growth is identified with the formation of a spatial polymer network. Consequently, to precisely analyze the changes caused by the injection, it was decided to perform three-interval thixotropic tests [54] in the oscillation-rotation-oscillation mode. In the rotation mode, the shear rate range encountered during the injection [28] was used.

**Figure 13:**
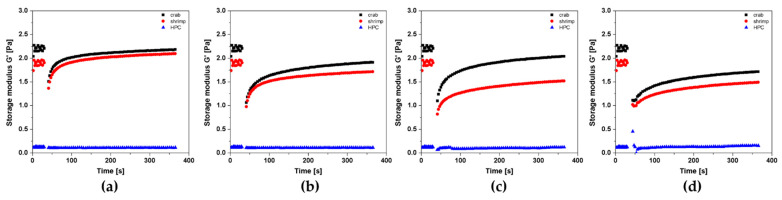
Kinetics of changes in the storage modulus G′ value over time during the 3ITT test depending on the applied shear rate in the second interval: (**a**) $\dot{\gamma }\;$= 600 s^−1^, (**b**) $\dot{\gamma }\;$= 2000 s^−1^, (**c**) $\dot{\gamma }\;$= 8000 s^−1^, (**d**) $\dot{\gamma }\;$= 15,000 s^−1^ for crab-derived chitosan (black), shrimp-derived chitosan (red), and HPC (blue) systems.

**Figure 14:**
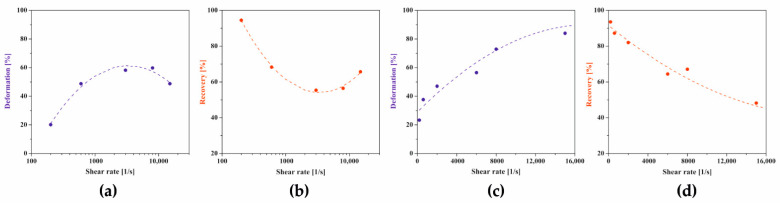
Dependence of changes in deformation (blue) and recovery (red) parameters on the shear rate in the second test interval for (**a**,**b**) crab-derived and (**c**,**d**) shrimp-derived chitosan systems.

Figure 13 shows the change in the course of the storage modulus G′ versus time for the characteristic values of the shear rate. It is visible that the highest values of the G′ modulus were obtained for crab-derived chitosan sol (G′ = 2.2 Pa). The chitosan sol obtained from the higher molecular weight polymer had slightly lower storage modulus values of about 2 Pa. It was found that in the case of chitosan systems, despite slightly different initial values of the dynamic modulus, after the rotational shear interval, both systems are characterized by almost the same elastic properties. The most significant differences are seen when measured with a shear rate of 8000 s^−1^. In this case, after applying the rotational shear interval, the storage modulus G′ values are higher for the polymer sol obtained from crab-derived chitosan. Based on the test results obtained, in particular the low values of the storage modulus of about 0.10–0.15 Pa, it was shown that the tested HPC solution has poor elastic properties and can store a small amount of energy in each deformation cycle. To quantify the changes in thixotropic properties caused by the varying shear rate in the second rotational interval, the deformation and recovery parameters determined for chitosan systems were analyzed (Figure 14). This analysis was not presented for the systems obtained from hydroxypropyl cellulose due to the almost unchanged elastic properties with simultaneous low values of the storage modulus, which could lead to a misinterpretation of the phenomenon.

In the case of crab-derived chitosan sol, it is visible that the course of both thixotropic parameters changes is again a non-monotonic dependence on the value of the shear rate. The use of the lowest shear rate of 200 s^−1^ in the rotational interval results in a slight deformation of 20% and the recovery of the structure in 95%. With the increasing shear rate, an increase in deformation is observed with a simultaneous gradual decrease in the degree of structure recovery. After reaching the extremum at $\dot{\gamma }$ = 3219 s^−1^ determined based on the maximum value of the approximating function, the deformation value decreases with a simultaneous increase in the degree of structure recovery 30 s after the end of the rotational interval. At the experimental point closest to the critical shear rate, i.e., for a shear rate of $\dot{\gamma }$ = 3000 s^−1^, both thixotropic parameters reach a value of about 58%. In the case of chitosan sol with a higher molecular weight, the change in the parameters of the three-interval thixotropic test was presented on a linear scale, similar to the dependence of the gelation time on the shear rate used in the initial interval (Figure 8b). It was found that the dependencies of both thixotropic parameters are quadratic functions of the applied shear rate in the second test interval. In the range of low shear rates, the determined parameters reach values similar to those calculated for the chitosan sol of lower molecular weight, i.e., the deformation reaches Def = 23% and the recovery Rec = 94%. With the increase of the shear rate used in the second test interval, the value of the deformation degree increases, while limiting the recovery of structure. After the highest shear rates (15,000 s^−1^) used had subsided, the deformation degree reached 81%, while the medium rebuilt its structure by 52%. Thus, in both cases, the obtained dependencies are fully consistent with the results determining the effect of the short-term rotation interval on the conditions of the sol–gel phase transition (Figure 4b and Figure 8b).

**Figure 15:**
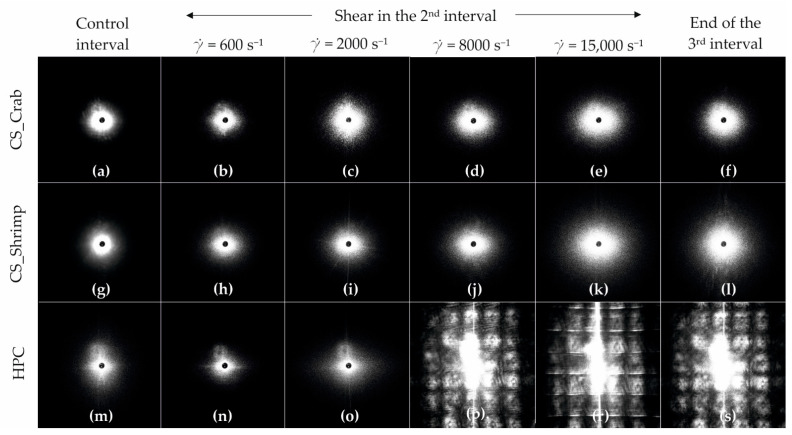
Recorded scattering patterns for the: (**a**–**f**) crab-derived chitosan, (**g**–**l**) shrimp derived chitosan, and (**m**–**s**) HPC systems at characteristic points of the 3ITT test, i.e., (**a**,**g**,**m**) control interval, (**b**–**e**,**h**–**k**,**n**–**r**) the first scattering pattern after the 2nd shear interval with a given shear rate, (**f**,**l**,**m**,**s**) the last scattering pattern from the 3rd interval.

Figure 15 shows the scattering pattern obtained during the three-interval thixotropic tests. It can be seen that for both chitosan systems, the presence of a unidirectional shear field in the second test interval results in an anisotropic intensity distribution in both directions; the intensity of these changes increases with the increase of the shear rate in the second interval. The observed differences between the systems result from an abrupt change in the anisotropy of the intensity distribution and a significant increase in the scattering area. In the case of crab-derived chitosan, such a change already occurs at a shear rate of 2000 s^−1^ (Figure 15c), while for shrimp-derived chitosan it is observed at a shear rate of 15,000 s^−1^ (Figure 15k). In the last case, for a shear rate value below 15,000 s^−1^, deformation of whole biopolymer molecules is observed, the intensity of which does not increase as rapidly as in the case of crab-derived chitosan. It is extremely important that at the end of the test, in each of the analyzed cases, the intensity anisotropy disappears, and the obtained shapes of the scattering images are spherical. This means that the systems returned to their original state despite the application of an intense shear field.

Completely different patterns were obtained when examining the sol obtained from a cellulose derivative. In this case, no significant differences are observed between consecutive images up to a shear rate of 2000 s^−1^. When using higher shear rates, the obtained patterns are similar to those in studies on small dispersed systems under high shear conditions [55]. The light was scattered multiple times during the measurements, due to the appearance of many colloidal particles in the camera’s field of view. Moreover, unlike chitosan systems, after the shear field has receded, the HPC structure does not return to its pre-shear state.

## 3. Discussion

Colloidal polymer systems that undergo thermoinduced sol–gel phase transition are considered to be very promising materials that can be used as injection drug carriers or matrices supporting the tissue regeneration process. The most common requirements for this group of materials, apart from their biodegradability and biocompatibility, are limited to remaining in the form of a sol before injection, as well as gelation under physiological conditions, with particular emphasis on temperature. Based on HPC studies, it has been shown that providing a liquid form of the sol may prove insufficient, especially when trying to use small injection needles (Figure 2), which would reduce the invasiveness of the administration. A similar non-injectable effect may occur with longer needles where the flow will result in greater pressure losses [28]. Thus, it seems reasonable to carry out instrumental injectability tests each time injection systems are designed.

**Scheme 1:**
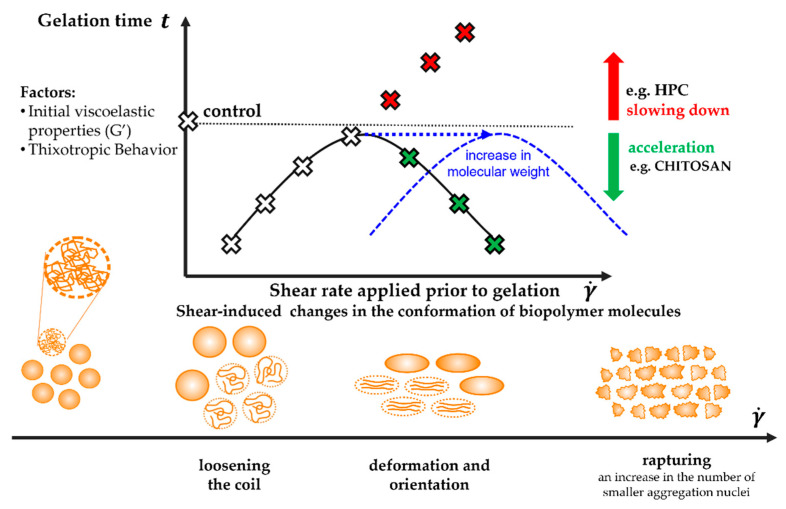
The proposed interpretation of the influence of the applied shear field on the conditions of the sol–gel phase transition conditions caused by changes in the conformation of the polymer molecules.

It has been shown that the flow of the polymer sol under the conditions of high shear rates observed during injection causes a significant increase in shear stress, which consequently affects the sol–gel phase transition conditions. However, this effect is neither dependent on a single parameter, nor is it a purely linear function of the applied shear rate. Based on the conducted research, three critical areas of the influence of flow conditions, i.e., shear rate, on the gelation kinetics, were determined and presented in Scheme 1.

Regardless of the tested system and the method of shear in the initial interval, the use of low, in the context of injection, shear rates of approx. 200 s^−1^, corresponding to the flow through the 14 G needle, significantly shortens the time necessary to observe the gelation characteristic point. Acceleration of the structure formation will most likely result from the synergistic effect of molecular, diffusion, and thermal changes. Under the influence of a short-term shear pulse, the polymer coils will loosen, and thus the access to the junctions zones will be facilitated [32]. This phenomenon, observed as an increase in the size of the molecules, is particularly visible for the crab-derived chitosan sol by reducing the light scattering area (Figure 15b), which, according to the scattering theory, indicate a larger colloidal particle size [51,56]. Moreover, due to the shear-thinned nature of the test medium the apparent viscosity of the system will decrease, thus the diffusion resistance will decrease, and consequently, the movements of aggregates will be intensified. Eventually, during the initial shear interval, an additional amount of mechanical energy will be supplied to the system, which will be converted into heat by dissipation, thus reducing the necessary amount of energy supplied to the system during storage at 37 °C. This thesis seems to be confirmed by changes in the initial values of the storage modulus G′ as a function of shear rate, particularly well visible in the case of crab-derived chitosan. It was found that the application of low shear rates increases the value of the G′ modulus by about 25% compared to the control measurement. It is worth noting that this parameter not only quantifies the elastic properties of the medium but is a measure of the energy stored in the system.

The acceleration of the sol–gel phase transition for both systems may also result from shear-induced precipitation of the polymer from the solution. Based on rheological studies combined with the SANS scattering technique carried out for the von Willebrand factor, it was shown that an increase in the rate above 2300 s^−1^ leads to the exposure of hydrophobic domains in the protein; the observed changes are irreversible [57]. In analogy to the flow-induced crystallization of the synthetic polymer (PEO) [37], in the case of the HPC-water system, a moderate shear field may break the protective water sheath surrounding the polymer chains [58]. While according to Supper’s theory [59], in the case of thermosensitive colloidal chitosan systems with the glycerophosphate salts, the addition of a polyol salt protects the polymer chain from precipitation at neutral pH by forming a protective shell. This prevents the formation of hydrophobic bonds, the formation of which is indicated as one of the mechanisms of thermoinduced sol–gel phase transition [60,61]. Therefore, it seems that under the influence of shearing the protective sheath may break and lead to the creation of aggregation nuclei [32,50].

As the shear rate increases, the time required for gelation to occur becomes longer. This is due to the progressive deformation of polymer molecules along the streamline, illustrated in the form of anisotropic intensity distributions of scattered light along two perpendicular directions (Figure 15b–e,h–k). Quantitative parameters confirming this thesis are changes in the value of deformation and the recovery determined based on the results of 3ITT tests. As shown in Figure 14, both thixotropic parameters reach the value of approx. 60% in the range of moderate shear rates observed during the injection. This means that proper aggregation must be preceded by relaxation of the stresses. Therefore, it can be assumed that the latter step will slow down the gelation process compared to the application of a short-term low-intensity shear interval. On the other hand, the kinetics of the sol–gel phase transition is still influenced by the phenomena described above, such as the reduction of the diffusion resistance due to the decrease in the fluid viscosity.

It would seem that a further increase in the value of the shear rate used in the initial shear interval would intensify the phenomenon of deformation of polymer molecules and further delay the phase transition. Such a phenomenon was observed for the thermosensitive sol obtained from cellulose derivative. Based on the analysis of the obtained light scattering patterns, it can be concluded that biomolecules are fragmented induced by mechanical stimuli, analogous to mechanical degradation caused by a short-term intense compression, tension, and/or shear [62,63,64]. Similar phenomena have been thoroughly described for protein systems. In the case of studies with the use of the von Willebrand factor in the shear rate range 190–4761 s^−1^, the presence of a larger number of small multimeters indicating progressive fragmentation was found [65]. Moreover, in studies with β-lactoglobulins, it has been shown that both continuous shear, as well as a short-term shear pulse, result in the production of fibers formation [66]. Wherein, a significant difference was observed in the distribution of the length of the produced filaments, i.e., continuous shear ensured a greater monodisperse. If the occurring fragmentation is irreversible, due to the limited ability to rebuild the structure, the time necessary to observe the critical point of the phase transition will be significantly longer (in the case of aggregation in the orthokinetic regime (Figure 11)) or even impossible to achieve in the case of aggregation induced solely by stochastic Brownian motion (Figure 12). In addition to the thixotropic properties, the initial viscoelastic properties of the tested medium seem to be important, with particular emphasis on the elastic properties, as well as the direct gelation mechanism. In the case of the sol obtained by dissolving hydroxypropyl cellulose in water, the storage modulus G′, identified with the properties of the polymer network, reached the values of approx. 0.10–0.15 Pa. This means that the spatial structure of the research medium is very poor and as a result of intense shearing ($\dot{\gamma }$ > 3000 s^−1^) it is further weakened.

Different behavior is observed in the case of chitosan systems. As it has been shown in the case of studies conducted for crab-derived chitosan sol, after reaching the 60% degree of deformation and recovery at the shear rates of approx. 3200 s^−1^ (Figure 14a,b), the characteristic time is shortened (Figure 7). It seems that similar to HPC, mechanical degradation of molecules is observed under the influence of an intense shear field. However, in this case, after the expiration of the short-term intense trigger, the system can form a spatial structure due to better viscoelastic properties and significantly higher initial values of the storage modulus G′. The shortening of the gelation time may result from the progressive fragmentation leading to the reduction of the size of the molecules with a simultaneous increase in their number. Consequently, a significant increase in the available junction zones and aggregation nuclei is observed. In addition, due to the smaller size, confirmed by rheo-optical tests, the transport of molecules in the continuous phase will be easier both by diffusion (perikinetics) and convection (orthokinetics). This concept is consistent with Alexander-Katz and Netz’s theory [67] concerning the instability of collapsed polymers under the influence of the shear field. According to the theory proposed based on numerical simulations at the molecular level, the unfolding of polymer chains will occur as a result of the formation of a protrusion, which will then refold while reducing the size of the molecules. This phenomenon will be observed when applying critical shear forces that will dominate the interactions between the polymer chains and the cohesive forces. A similar concept of shear-induced structural changes has been proposed for proteins in which the tertiary structure would be destroyed [47]. In both of the above-mentioned issues, the influence of, e.g., molecular weight and conformation of polymer chains is still controversial.

Based on the test results obtained for both chitosan systems, it can be seen that the molecular weight of the polymer is a parameter that significantly affects the value of the critical shear rate, above which fragmentation of molecules may occur. Regardless of the aggregation regime and the type of initial shear interval, in the case of using chitosan of crab origin, the value of the critical shear rate was approx. 3200 s^−1^, while when using chitosan with a higher molecular weight (of shrimp origin) it increased to approx. 8000 s^−1^ (during shearing in the needle) and 19,200 s^−1^ (shearing directly in the measuring system of the rheometer). It should be noted that the last of the reported values was determined based on extrapolation of the approximating function of the experimental data and exceeds the range of applicability of the rheometer measuring system used. Due to the similar size of both molecules, determined based on the rheo-optical studies, it should be assumed that the chitosan chains with a higher molecular weight are much more tightly packed, and consequently, it is possible to create more bonds between the functional groups. Consequently, the higher molecular weight system has stronger cohesive properties, which can only be dominated by using significantly higher shear forces than the lower molecular weight polymer. Additionally, for a higher molecular weight polymer, the type of initial shear interval used is much more important. Despite the satisfactory consistency of the obtained results for crab chitosan sols, it was shown that when the initial shear interval is applied directly in the rheometer measuring system, the model curve has a more flattened shape, which suggests a less invasive measurement and thus a less impact on the gelation conditions. The lack of reaching the extreme value of the gelation time at the critical value of the shear rate in the case of the shrimp-derived chitosan seems to confirm the lower invasiveness of the shear carried out directly in the rheometer measuring system.

The last, extremely important aspect that can explain the differences in the gelation conditions preceded by shear in both tested polymers may result primarily from interactions at the molecular level and different mechanisms of forming an unlimited polymer network. It is known that in the case of purely physical thermoinduced sol–gel phase transition, an example of which is HPC gelation [19], the gel produced is much less stable, and the process itself is fully reversible, unlike the two-stage aggregation of chitosan systems, during which diffusion-limited aggregation is preceded by reaction-limited aggregation [32,68].

It was unequivocally demonstrated that the initial shear interval significantly affects the conditions of the sol–gel phase transition of polymer systems. Moreover, from the administration point of view and the gelation conditions, the use of the needles with the largest diameter, e.g., 14 G–16 G, seems to be the most advantageous. Unfortunately, compared to thinner needles, the patient’s discomfort during application will be much greater, but still this administration method will be less invasive than the implant scaffolds. Based on the precise instrumental tests, both the injectability as well as the conditions of polymer structure formation after application, it can be noticed that the chitosan-based systems are an ideal injection matrix due to the possibility of administration with the use of thin needles and simultaneous rapid phase transition inside the human body. A practical aspect of the conducted research is the indication that the use of needles with small diameters without preliminary tests may lead to an extension of the phase transition time and, consequently, the spilling of the polymeric carrier before gelation. Finally, it was confirmed that maintaining a liquid form of the sol does not ensure the possibility of injection of a given system, and the determined times of the sol–gel phase transition using a rheometer are much shorter than in the case of aggregation at rest, e.g., in a heating chamber. In the extreme case, as shown for HPC, despite the occurrence of the phase transition point in the rheometer measurement system, aggregation without an imposed flow is impossible. This is due to different aggregation regimes, orthokinetics, and perikinetics, respectively.

The conducted research provides so far unpublished reports on the influence of the shear field on the conditions of thermoinduced sol–gel phase transition determined by the proposed direct and indirect research methods.

## 4. Materials and Methods

The studies used chitosan of crab (Sigma Aldrich, Poznan, Poland, product No. 50494) and shrimp (Sigma Aldrich, Poznan, Poland, product No. 50494) origin with different molecular weight, hydroxypropyl cellulose (Sigma Aldrich, Poznan, Poland, product No. 191884), hydrochloric acid (Fluka Analitycal provided by Alchem, Torun, Poland, product number: 84415), and disodium β-glycerophosphate (Sigma Aldrich, Poznan, Poland, product No. 50020).

### 4.1. Preparation of Thermosensitive Hydrogels

A total of 400 mg of chitosan was dissolved in 16 mL of 0.1 M hydrochloric acid solution and left for 24 h at room temperature. Subsequently, a suspension of disodium β-glycerophosphate (GP) was prepared by dispersing 2 g of the powder in 2 mL of distilled water. Then, both systems were cooled separately for 2 h at 4 °C to finally introduce the GP suspension drop by drop into the polymer solution. The obtained material, in accordance with the commonly used preparation [16], was stored in the refrigerator for another 24 h before the actual measurement.

Powdered hydroxypropyl cellulose was dissolved in distilled water to obtain 5 to 20% solutions, and then left for 48 h at room temperature to completely dissolve the polysaccharide. After carrying out the preliminary rheological characterization, the optimal concentration of the HPC solution equal to 10% was selected and the system was directed to the proper study of the injection influence on the phase transition conditions.

### 4.2. Rheological Analysis-Research Apparatus

Rheological tests were carried out with the use of an Anton Paar MCR301 (Anton Paar, Warszawa, Poland) rotational rheometer equipped with a cone-plate CP50-1 measuring system (cone diameter 50 mm, angle 1°, truncation 48 µm).

Rheological tests combined with the simultaneous small angle light scattering (SALS) analysis were carried out using an Anton Paar MCR502 (Anton Paar, Warszawa, Poland) rotational rheometer equipped with a dedicated glass measuring system PP43/GL-HT. The measurement gap was 0.3 mm.

### 4.3. Preliminary Rheological Characterization of Hydroxypropyl Cellulose Sol

In order to select an optimal concentration of HPC solution for proper measurements of the effect of injection on the sol–gel phase transition conditions, non-isothermal gelation kinetics were tested from 5 °C to 60 °C (heating rate 1 deg/min) at constant deformation (angular frequency ω = 5 rad/s and amplitude γ = 1%) [18,68,69].

In order to determine the rheological parameters of the polymer sol, flow curves in the range of shear rates of 100–15,000 s^−1^ were determined. The lower value of the range resulted from the possible values of the shear rate observed in the 14 G needle using the 1 mm/s crosshead speed; the upper value is due to device limitation. The measurement was performed at 20 °C.

Instrumental injectability tests were performed using a Brookfield CT3 texture analyzer (Brookfield, London, United Kingdom) with a cell load of 4.5 kg (44 N) in compression mode. In the study, injection needles (Zarys, dispoFINE) in sizes 14 G–25 G and 2 mL disposable syringes with an internal diameter of 9.75 mm (Braun Injekt) filled with 0.5 mL of the experimental medium were used. The tests were carried out with a typical manual injection speed of 1 mm/s [30,31], and the injection was carried out “into the air”. The system injectability was evaluated based on the dynamic glide force (DGF) value, i.e., the force necessary to maintain the syringe plunger movement. Its value was defined as an average of the longest range of forces in which they have a constant value [28,30,48].

### 4.4. Kinetics of the Phase Transition after Shearing

The kinetics of the sol–gel phase transition of the studied polysaccharide systems was investigated in the peri- and orthokinetic aggregation regime under isothermal conditions at 37 °C. The aggregation conditions in the perkinetic regime were determined using the TurbiscanLab device (Formulaction, Toulouse, France), while the aggregation conditions in the orthokinetic regime were determined using a rotational rheometer. Constant deformation (angular frequency ω = 5 rad/s and amplitude γ = 1%) was used in the tests [32,50].

**Scheme 2:**
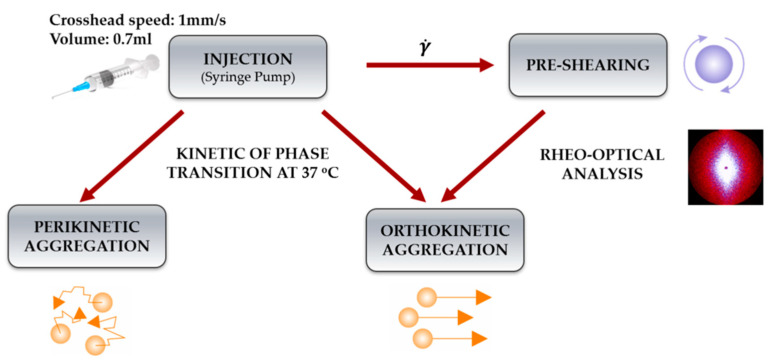
Illustration of the experimental setup of the shear effect on gelation conditions depending on the aggregation regime.

The initial shear interval preceding the actual measurements of the aggregation kinetics was performed in two ways. In the first one, the sample was sheared in the injection needle using a typical manual injection speed. A Harvard PHD2000 infusion pump was used in the tests to ensure a set, constant value of the injection rate. In the second case, used when testing the gelation conditions in orthokinetic aggregation, the sample was subjected to rotational shear for 3 s in the shear rate range of 200–15,000 s^−1^ directly in the rheometer measuring system. Scheme 2 shows an overview of the conducted experiments.

### 4.5. Rheo-Optical Analysis of Conformational Changes Caused by the Injection

In order to determine the effect of the shear interval on the structure of biomolecules on a macroscopic scale, three-interval thixotropic tests were performed in the oscillation-rotation-oscillation mode [28,54,68] combined with simultaneous small-angle light scattering analysis [51]. Constant deformation values of γ = 1% and ω = 5 rad/s lasting 30 s and 300 s, respectively, were used in the oscillatory shear intervals. The rotational shear interval lasted 3 s; the values of the shear rate were changed in the range of 200–15,000 s^−1^.

## 5. Conclusions

The conducted studies unambiguously reveal the influence of the shear field on the conditions of the sol–gel phase transition of polysaccharide colloidal systems, which has not been discussed in the literature so far. Based on the research carried out in the range of the shear rate from 200 s^−1^ to 55,000 s^−1^, two types of the observed changes have been demonstrated. The proposed research methodology allows determining the potential of the polymeric material as a minimally invasive drug carrier or cell scaffold, including instrumental assessment of the risk of extending the gelation time, which may lead to the sol spillage before in situ gelation.

It should also be noted that from the point of view of fluid mechanics and capillary rheometry of non-Newtonian fluids, the phenomena occurring during the injection will also be observed during the flow through the 3D printer’s nozzle. Therefore, similar test procedures should be carried out to reflect the conditions of print formation after the flow of the thermosensitive filament through the printer nozzle as well as to consciously design injection systems for biomedical applications.

## Data Availability

The data presented in this study are available on request from the corresponding author.
